# Tracking development assistance and government health expenditures for 35 malaria-eliminating countries: 1990–2017

**DOI:** 10.1186/s12936-017-1890-0

**Published:** 2017-07-14

**Authors:** Rima Shretta, Brittany Zelman, Maxwell L. Birger, Annie Haakenstad, Lavanya Singh, Yingying Liu, Joseph Dieleman

**Affiliations:** 1 Global Health Group, University of California, San Francisco, 550 16th St, 3rd Floor, Box 1224, San Francisco, CA 94158 USA; 2 Swiss Tropical and Public Health Institute, Socinstrasse 57, 4002 Basel, Switzerland; 3 University of Basel, Petersplatz 1, 4001 Basel, Switzerland; 4 Columbia University College of Physicians and Surgeons, New York, USA; 5 Harvard T.H. Chan School of Public Health, Boston, USA; 6 Duke University Sanford School of Public Policy, Durham, USA; 7 University of Kentucky, Lexington, USA; 8 Institute for Health Metrics and Evaluation, University of Washington, Seattle, USA

**Keywords:** Malaria, Elimination, Financing, Development assistance for health, Government health expenditure, Domestic expenditure

## Abstract

**Background:**

Donor financing for malaria has declined since 2010 and this trend is projected to continue for the foreseeable future. These reductions have a significant impact on lower burden countries actively pursuing elimination, which are usually a lesser priority for donors. While domestic spending on malaria has been growing, it varies substantially in speed and magnitude across countries. A clear understanding of spending patterns and trends in donor and domestic financing is needed to uncover critical investment gaps and opportunities.

**Methods:**

Building on the Institute for Health Metrics and Evaluation’s annual Financing Global Health research, data were collected from organizations that channel development assistance for health to the 35 countries actively pursuing malaria elimination. Where possible, development assistance for health (DAH) was categorized by spend on malaria intervention. A diverse set of data points were used to estimate government health budgets expenditure on malaria, including World Malaria Reports and government reports when available. Projections were done using regression analyses taking recipient country averages and earmarked funding into account.

**Results:**

Since 2010, DAH for malaria has been declining for the 35 countries actively pursuing malaria elimination (from $176 million in 2010 to $62 million in 2013). The Global Fund is the largest external financier for malaria, providing 96% of the total external funding for malaria in 2013, with vector control interventions being the highest cost driver in all regions. Government expenditure on malaria, while increasing, has not kept pace with diminishing DAH or rising national GDP rates, leading to a potential gap in service delivery needed to attain elimination.

**Conclusion:**

Despite past gains, total financing available for malaria in elimination settings is declining. Health financing trends suggest that substantive policy interventions will be needed to ensure that malaria elimination is adequately financed and that available financing is effectively targeted to interventions that provide the best value for money.

## Background

The launch of the Roll Back Malaria Partnership (RBM) in 1998 and the Millennium Development Goals in 2000 catalysed unprecedented political and financial commitment for malaria from donors, such as the Global Fund to Fight AIDS, Tuberculosis and Malaria (Global Fund), the US President’s Malaria Initiative (PMI), the World Bank, and others as well as endemic countries themselves. As a result, global malaria incidence and deaths have dramatically declined by 37 and 60%, respectively, between 2000 and 2015 [1]. Between 2000 and 2015, 17 countries have eliminated malaria, six of which have been certified as malaria-free by the World Health Organization (WHO) [1]. Thirty-five countries are currently actively pursuing malaria elimination, with elimination goals ranging from 2016 to 2035 [2]. According to WHO, 21 countries are in a position to achieve at least one year of zero indigenous cases of malaria by 2020 [3].

Despite this unprecedented progress, donor funding for malaria has declined since 2010 and is projected to continue to decline [4, 5]. These reductions in external financing are even greater for the sub-set of malaria eliminating countries despite demonstrated evidence on the returns on investment from elimination [6]. By nature, these countries have lower disease burdens and are often lower-middle or middle-income countries and therefore a lesser priority for donors [5].

The Global Fund, which has been the largest external financier supporting eliminating nations, has historically dispersed about 7% of its total portfolio to eligible malaria-eliminating countries. However, under the New Funding Model adopted in 2012, resources for this sub-set of countries declined to less than 5% [5] and have declined further under a revised allocation-based model adopted by the Global Fund Board in November 2016 [7]. Other bilateral and multilateral donors are similarly diverting resources to higher-burden countries with the least ability to pay as measured by their Gross National Income (GNI) [8, 9]. In some cases, donors are entirely moving away from disease-based funding to general system strengthening to address concerns of global health security [10]. While integrated systems might help countries in the final push to malaria elimination and prevent reintroduction of malaria, a well-funded malaria programme, maintaining a level of vertical oversight, is crucial in the short to medium term [10]. At the same time, as the disease becomes less “visible”, government funds for malaria are often diverted to other health priorities that are perceived to be greater health threats, risking a reversal of the recent gains made in malaria elimination [11].

Reductions in financing for countries eliminating malaria comes at a critical time—WHO’s Global Technical Strategy (GTS) for Malaria 2016–2030 and the Roll Back Malaria Partnership’s Action and Investment to Defeat Malaria 2016–2030 (AIM) together with the recently endorsed Sustainable Development Goals, set their sights on rapid progress with malaria elimination towards attainment of malaria free status in 35 countries by 2030. Total funding for malaria control and elimination was estimated at $2.9 billion in 2015 [1], representing just 46% of the GTS 2020 milestone of $ 6.4 billion. Achieving the global goals will require sustained financial and political commitment at the global and domestic levels [2]. The investments have the potential to deliver strong health benefits through fewer deaths and less illness valued at over $49 billion, exceeding investment costs by a factor of 40 between 2015 and 2030.

There is little published information about the international resources funding malaria elimination efforts, how these funds are spent and their association with domestic financing. Several published studies describe disbursements of development assistance for health (DAH) and government health expenditure (GHE). The Institute for Health Metrics and Evaluation (IHME) [12] has been tracking DAH from 1990 onwards, disaggregating spending by the source of funding, intermediary channel, recipient country, and health focus area. Some studies have concentrated on specific health focus areas, such as HIV and the estimates produced by Countdown to 2015 [13], which focused on maternal, child and newborn health. WHO annually publishes a World Malaria Report [3], which includes government expenditure information obtained from countries’ national malaria control programmes. However, expenditure data are often unavailable and replaced by budget information. Pigott et al. [14] collated co-financing data from the Global Fund grant proposals to obtain government budgets on malaria interventions. The system of national health accounts, available in a limited number of countries, provide valuable information about financing flows, but are limited by issues of comparability, timeliness and level of reporting. Past analyses have either focused on single countries and/or disease programmes or across multiple countries aimed at measuring the effectiveness of DAH by exploring how DAH is allocated across recipient countries and/or health focus areas or interventions.

To better understand past and future trends in financing for malaria elimination, this paper systematically tracks malaria-specific estimates of DAH expenditures from all major international development agencies from 1990 to 2013 with projections up to 2017, and splits this spending into 13 malaria activities or intervention areas that describe how the resources were used. In addition, GHE as a source for malaria financing was tracked from 2000 to 2014 to explore associations between DAH and GHE to inform future decision-making and better align need with actual resource allocation. A clear perspective on where resources have been and will be available will uncover critical investment gaps and investment opportunities.

Specifically, the paper aims to: (a) track development assistance for the prevention and treatment of malaria from channel to recipient country or region, for 1990–2013s; (b) generate lower-bound estimates of how development assistance for the prevention and treatment of malaria was used by activity or intervention area for the same time period; (c) estimate GHE for malaria from 2000 to 2014; and, (d) estimate DAH projected financing from 2014 to 2017 in the 35 eliminating countries.

## Methods

**Fig. 1:**
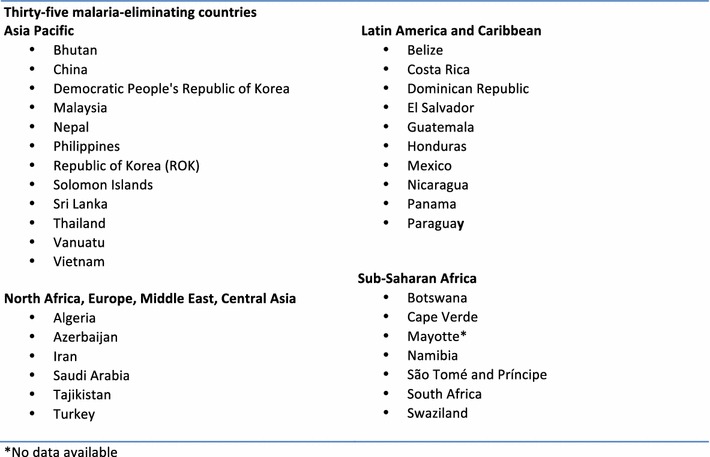
List of malaria-eliminating countries included in this analysis

This analysis was conducted in 35 malaria-eliminating countries defined in 2015 as countries that have a national or sub-national evidence-based elimination goal and/or are actively pursuing elimination (zero malaria transmission) within its borders [15] (see Fig. 1).

### DAH

DAH is defined as the financial and in-kind contributions for maintaining or improving health in low- and middle-income countries. This analysis focuses on financial contributions, as there is no reliable database that captures in-kind contributions. Disbursement of development assistance for malaria was estimated to the 35 countries for 1990 through 2013. Building on the IHME’s annual Financing Global Health research, data were collected from primary agencies and organizations that channel DAH or third party organizations or private organizations that collect such data [12]. Detailed methodology is described elsewhere [16], however, in brief, resources were tracked from the channel back to the source (original donor) where possible, and further forward to the country or region recipient. This permits disaggregation of data into categories such as private or specific public sources, bilateral and multilateral agencies, and recipient countries. When underlying disbursement data were not available, disbursements were estimated using econometric time-series methodologies and appropriations or commitment data. Double counting generated by transfers among channels was removed manually in order to estimate a total envelope without exaggerating the true amount of resources provided. Throughout this analysis, figures are standardized to US$ 2014 to allow for uniform comparisons.

### DAH by service delivery area

DAH for malaria elimination was split into categories identifying the type of investment. The Organization for Economic Cooperation’s (OECD) Creditor Reporting System (CRS) database contains information on DAH that has been channeled through bilateral agencies [17]. From the CRS data, the amount of DAH disbursed per project, the recipient country, the project title, and the project description was collated. A keyword search was run to further disaggregate malaria DAH into intervention or activity categories. For Global Fund malaria grants, budget data were extracted by service delivery areas from programme grant agreements. The fraction of aid allocated to every service delivery area for each year in a grant was calculated, and the budgeted malaria aid fractions to actual DAH for each year of a grant were applied. When budget information was missing from a programme grant agreement, DAH was distributed to the service categories based on service delivery areas that were listed in the Global Fund online grants portfolio for the specific grant. Some funders, such as the World Bank, did not have this kind of information and therefore, funding by service delivery areas was unable to be disaggregated.

### GHE

A diverse set of data points and reports were used to estimate the share of domestic government health budgets spent on malaria from 2000 through 2014. The WHO annually publishes a World Malaria Report (WMR), which includes government expenditure (or budget information when expenditures are unavailable) obtained from countries’ national malaria control programmes. GHE as source data were extracted from these reports from 2008 to 2015 and from Pigott et al. [14], which collated co-financing data from the Global Fund grant proposals to obtain government budgets on malaria treatment. Each data source has its own concerns. Government expenditure published in the WMR does not generally provide comprehensive tracking of spending on healthcare workers and capital costs. In addition, reports from different years are inconsistent, mostly due to weak or non-existing expenditure tracking systems, impeding any temporal comparisons. Pigott et al. reports government expenditure that includes spending on human resources, but these numbers are from government budgets rather than actual expenditure. If budgets and spending differ in a non-random manner these estimates will be biased. To estimate government expenditure that is comprehensive of all public spending on malaria, a linear regression on data from both sources was performed. Country-specific regression analyses took into account country, the year the data were published, whether the data were comprehensive of human resources and capital costs, whether the data were expenditure or budget, and time. These were modelled using basis splines to avoid assuming linear growth.

### Estimates of DAH projected financing from 2014 to 2017

To estimate projected DAH spending, a regression that took into account DAH averages to recipient countries and budgeted or earmarked funding was used. The dataset used to train the model was tailored to reflect the data available for each forecast. These individual training sets were made in order to take into account future malaria projects for which financial commitment data was not available at the time of writing this paper.

### Uncertainty estimates

Uncertainty intervals for government health expenditure and DAH projected financing from 2014 to 2017 were calculated by sampling the variance–covariance matrix generated by each linear regression 1000 times.

### GHE as a function of GDP and disease burden

To assess the association between GHE and a country’s income as measured by the Gross Domestic Product (GDP) per capita, GHE for malaria as a percentage of total health expenditure was plotted against GDP and further analysed by malaria disease burden as measured by Annual Parasite Index (API).

## Results

### Funding landscape for malaria elimination

**Fig. 2:**
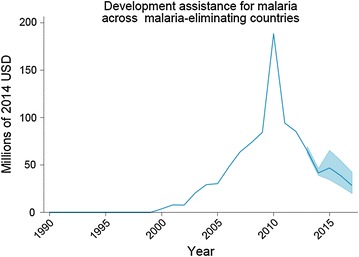
Donor assistance for health (DAH) past and future projections for 35 malaria eliminating countries

Between 2000 and 2010, the overall funding for malaria for the 35 malaria-eliminating countries grew 2.5-fold from $179 million in 2000 to over $458 million in 2014. Despite a reduction in overall funding after 2010, total funding to these countries amounted to over $335 million in 2013 of which 81% was from domestic resources and 19% from donors. South Africa was later excluded in subsequent analysis as it had significant GHE for malaria until 2009, thereby skewing the results of the underlying trend in GHE by the remaining 34 countries. Without South Africa, total financing amounted to $430 million in 2014 (see Fig. 2).

### DAH

**Fig. 3:**
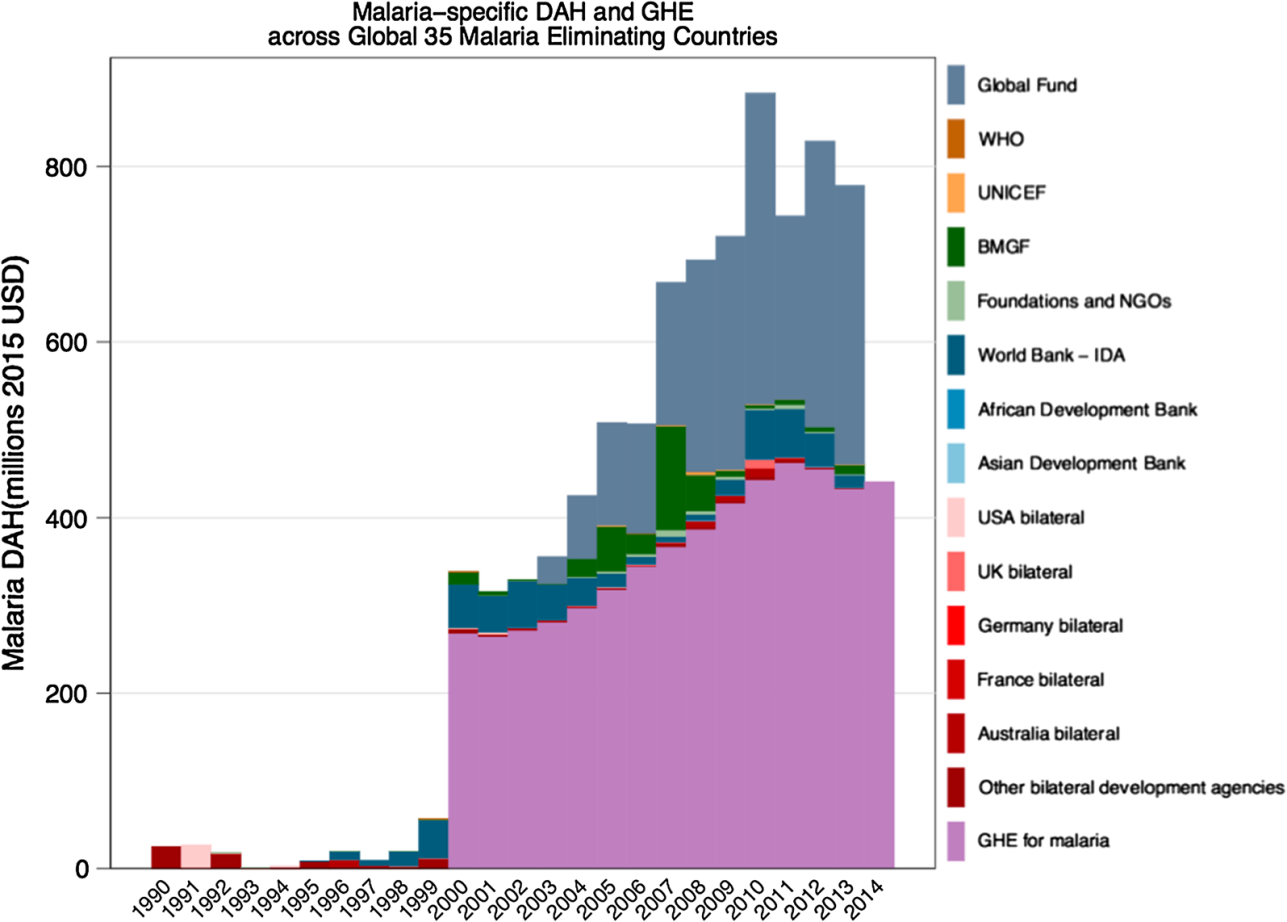
Donor assistance for health (DAH) and Government health expenditure (GHE) by funding channel graph for 34 countries (excluding South Africa). GHE data only available after 2000

DAH increased 33-fold between 2000 and 2010 for the 35 malaria-eliminating countries from just over $5 million in 2000, accelerating after 2007, and peaking at over $176 million in 2010. However, DAH sharply declined by over 65% between 2010 and 2013 to about $60 million. The largest declines in DAH were seen in China which was 90% externally financed in 2010 compared to only 10% in 2013 and in Democratic People’s Republic of Korea and the Solomon Islands with declines of over 25%. Nonetheless, external funding was 11.5-fold higher in 2013 than in 2000. In 2014, DAH accounted for less than 10% in Azerbaijan and Belize. Overall financing trends are projected to continue to decrease between 2014 and 2017 with a low of $28 million in 2017 (uncertainty interval $9.6 million to $66.4 million). Figure 3 illustrates malaria expenditure by donors (by the primary sources or intermediary channels) from 1990 and projected to 2017, and government from 2000 (when data was available from) for the 34 malaria-eliminating countries (excluding South Africa).

The Global Fund was the largest source of external funding for malaria-eliminating countries providing 96% of the total DAH in the 35 countries in 2013. However after peak funding in 2011, Global Fund resources for these countries decreased by approximately 58% from over $140 million in 2011 to approximately $60 million in 2013. Other donors that provided funding to malaria-eliminating countries over the period 2007–2011 included the World Bank, the Australian government (particularly for the Pacific islands), and the Bill & Melinda Gates Foundation (BMGF). Malaria-specific funding from the World Bank halted in 2012 with the conclusion of the World Bank Booster Programme for Malaria. Similarly bilateral funding from Australia decreased sharply in 2011 by 64% decreasing further with the integration of Australia’s aid programme into the Department of Foreign Affairs and Trade.

### DAH by service delivery area

**Fig. 4:**
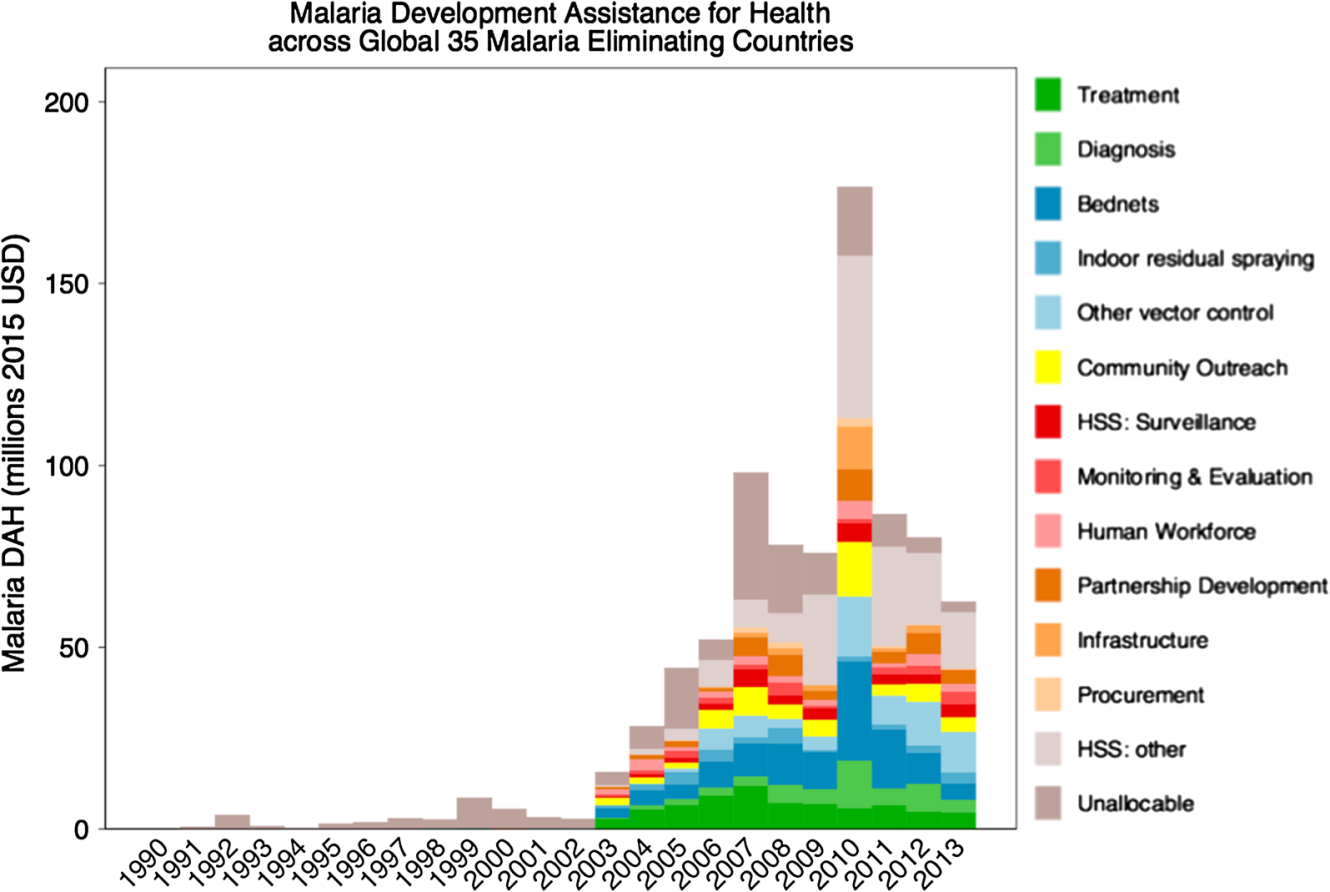
Donor assistance for health (DAH) by service delivery area for 35 countries

Figure 4 illustrates the trend in spending by service delivery area in the 35 malaria-eliminating countries. The graph indicates that DAH channels prioritise various service delivery areas at different times. In general, DAH increased along all interventions starting in 2003 and peaking in 2010 at over $176 million. Treatment, diagnosis and vector control [indoor residual spraying (IRS) and bed nets], and to a lesser extent, health system strengthening and surveillance grew at faster rates than other service delivery areas, consistent with recommendations for malaria elimination. Exceptions included the Dominican Republic where surveillance accounted for 40% of expenditures in 2009 declining to less than 10% in 2013. Expenditures for malaria treatment increased between 2003 and 2007 but have declined since 2010. At the same time, DAH expenditures on diagnosis increased gradually, consistent with WHO recommendations on testing before treatment, peaking in 2010, but decreasing thereafter. In most countries, the ratio of DAH expenditure on diagnosis versus treatment increased after 2008, reaching a 50:50 split in Bhutan and Costa Rica by 2013. A notable exception is Thailand with 25% of total expenditure on treatment but very little on diagnosis. There was a high growth in vector control spend particularly on bed nets as well as other undefined vector control interventions peaking in 2010 and declining thereafter. By 2012, expenditures on bed nets were less than other vector control interventions. However, bed nets still accounted for 80% of expenditure in Bhutan. Other vector control interventions accounted for over 80% of total expenditure in Nepal, and up to 50% in Sao Tome and Nicaragua. There was some growth in community outreach and strengthening of surveillance systems, however, this growth was not uniform; with surveillance expenditure actually decreasing overall between 2010 and 2012. A large proportion of funds could not be allocated over any of the service delivery areas particularly between 2008 and 2011 (14%), specifically in Indonesia and India.

### GHE for malaria

For the 35 malaria-eliminating countries in aggregate (excluding South Africa as an outlier), GHE as source for malaria elimination steadily increased since 2000 from about $131 million per year to about $250 million in 2014, outpacing DAH. In 2010, at the peak of external finding, government spending was 1.4 times higher than the donor resources available.

Table 1 shows the growth rates across various time periods for both GHE and DAH for the 35 malaria-eliminating countries.

**Table 1:** DAH and GHE annualized growth rates for the 35 malaria eliminating countries

Countries	2000–2004		2005–2009		2010–2013 or 2014	
	DAH	GHE	DAH	GHE	DAH (through 2013)	GHE (through 2014)
	−42.2	11.1	116.0	11.2	74.7	−25.8
Algeria	0	1.3	0	−0.2	0	−74.7
Azerbaijan	−100.0	7.4	306.7	27.8	−19.4	7.2
Belize	0	2.3	0	9.9	0	4.9
Bhutan	−100.0	4.1	2.8	−0.7	−6.9	−2.7
Botswana	0	−6.1	0	29.3	−100.0	9.1
Cape Verde	0	3.5	−100.0	7.7	0	−15.9
China	51.2	6.2	49.8	31.0	−67.4	37.6
Costa Rica	0	2.3	0	7.3	0	−9.2
Democratic People’s Republic of Korea	−100.0	8.2	−100.0	5.9	−31.5	−0.2
Dominican Republic	0	−26.3	0	15.5	−2.5	3.6
El Salvador	0	−9.5	0	31.8	0	14.2
	0	−2.0	0	−0.2	0	−22.2
Guatemala	0	15.9	−29.7	17.2	0	−46.0
Honduras	176.0	−1.3	−14.5	−12.5	−13.2	−14.5
Iran	−100.0	7.5	18.2	4.8	10.7	−15.1
Malaysia	0	−0.7	0	16.5	0	10.8
Mayotte^a^	–	–	–	–	–	–
Mexico	0	0.3	0	3.0	0	−6.7
Namibia	92.0	11.9	24.5	−0.6	43.2	5.2
Nepal	65.0	4.5	42.3	1.6	−22.2	14.0
Nicaragua	0	−4.9	21.4	−7.1	3.8	−17.2
Panama	0	−23.0	0	17.0	0	38.5
Paraguay	0	1.5	0	11.2	0	13.1
Philippines	74.1	−12.3	−17.3	50.4	−43.7	18.0
Republic of Korea	0	−2.2	0	13.5	0	−13.4
Saudi Arabia	0	2.0	0	7.6	0	−2.3
Solomon Islands	78.2	11.2	56.3	37.8	−45.7	−27.9
South Africa	−12.9	5.1	−34.7	−1.8	31.6	−5.9
Sri Lanka	101.8	13.0	53.2	1.6	−12.8	−34.4
Swaziland	0	−1.5	74.4	8.6	−2.7	−8.9
São Tomé and Príncipe	6.3	2.0	−49.9	28.6	49.1	37.1
Tajikistan	−54.1	5.0	99.0	21.2	−12.2	32.2
Thailand	−17.2	−5.6	−7.2	−14.3	46.6	6.5
Turkey	0	79.5	0	12.8	0	−2.9
Vanuatu	0	−0.5	−25.5	6.6	−11.6	−8.4
Vietnam	61.1	−0.2	8.6	0.4	−46.0	−11.5
Total	0.6	2.6	1.6	2.9	−0.2	3.1

^a^No data available

### GHE as a function of GDP and API

**Fig. 5:**
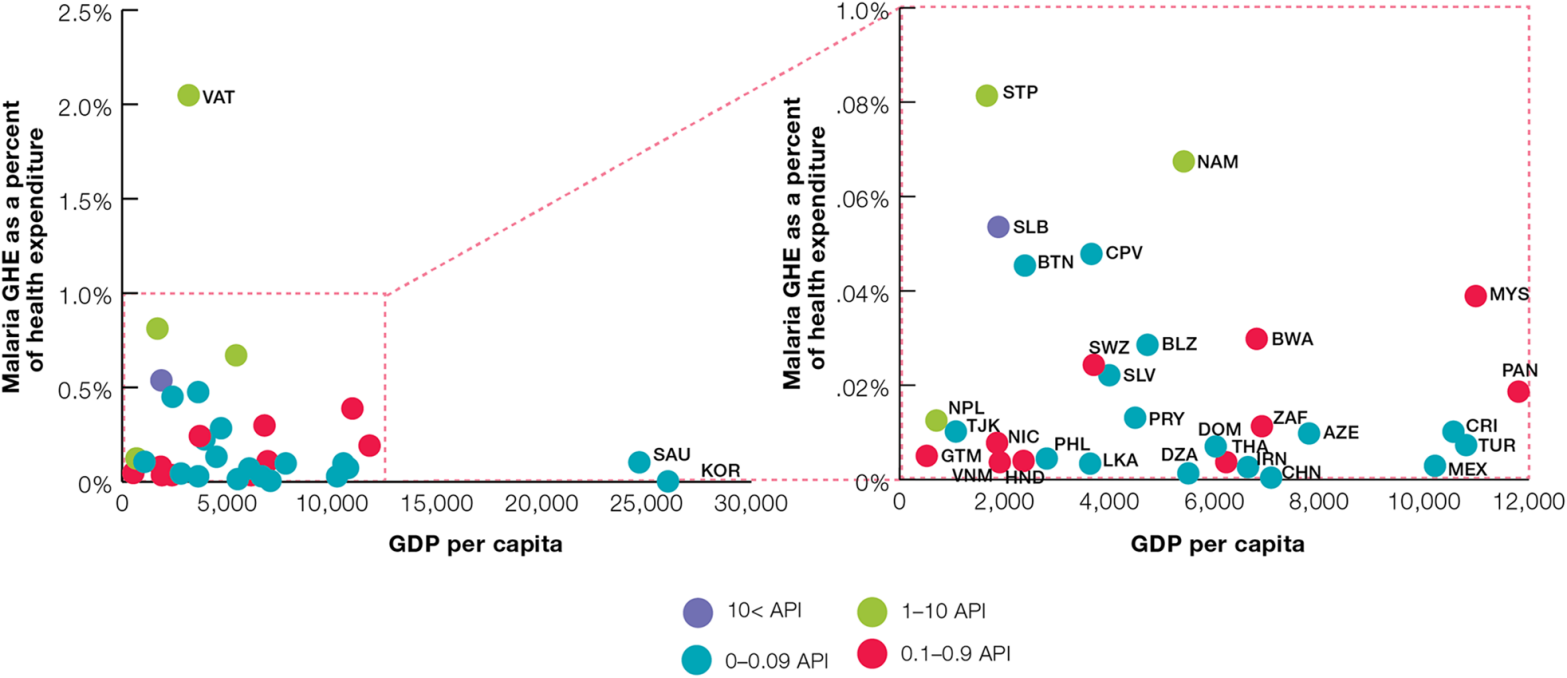
GHE for malaria as a per cent of health expenditure by GDP and API

Figure 5 illustrates government health expenditure for malaria as a function of GDP and API. There is a wide variation in the GHE on malaria uncorrelated with GDP indicating that GDP is not directly associated with increased domestic spending in malaria. Higher GDP countries with low government expenditure on malaria include several countries in Latin America (Costa Rica, Panama, Belize) as well as Swaziland and Thailand. Most of the countries spent less than 0.05% of their GDP on malaria with the exception of Vanuatu (0.1%). Furthermore, the Figure illustrates that malaria expenditure is also not directly associated with disease risk as measured by API.

## Discussion

This is the first study that tracks DAH and GHE specifically for malaria eliminating countries from 1990 to 2014 with projections to 2017. This study also makes use of enhanced methods providing a more comprehensive tracking of DAH and GHE than has previously been utilized in other studies. The findings clearly demonstrate a growing uncertainty about the future availability of DAH for malaria elimination. At the same time, while government health expenditures have steadily increased, they have not kept pace with the declining DAH. Many malaria-eliminating countries could risk facing significant funding gaps, which can increase the risk of malaria resurgence highlighting the need for an interim solution until the economies of these countries have sufficiently grown to fill the gap.

The findings demonstrate three periods for DAH for malaria: a period of moderate growth in the 1990s, accelerated growth in the first decade of the 2000s of 97%, and a decline of 65% since 2010. In the 35 countries included in this review, total financing for malaria grew from $179.5 million to $301.7 million between 2000 and 2013 of which DAH accounted for 19% in 2013. DAH began to decline in 2011, coinciding with the Global Fund’s decision to halt its 11th grant cycle. During this period, DAH declined by 65% in the 35 malaria-eliminating countries overall and is projected to further decline through 2017.

The new allocation methodology adopted by the Global Fund in 2012, uses a combination of disease burden and GNI per capita to determine the financing that countries will receive for the three diseases. Under this New Funding Model, country-specific funding to the sub-set of countries attempting to eliminate malaria has declined by over 30% [5]. Further declines in allocations have been noted under a revised model adopted in November 2016. These changing polices have major implications for the financing and delivery of health services, particularly for malaria elimination. Eliminating countries typically have lower disease burdens and are often middle-income countries and therefore tend to be less attractive investments for donors looking for easy to measure high impact results. Of the 35 countries included in this review, 2 are high-income countries, 15 are upper middle income, 14 are lower middle income, and 3 are lower income (no data was available on Mayotte). Eighteen of these countries are ineligible to receive Global Fund financing. Three countries have graduated from Global Fund malaria financing in the past 6 years: China (2011), Dominican Republic (2013) and Iran (2012) and one country transitioned out of Global Fund support in 2016 (Paraguay). Sri Lanka, which attained malaria-free certification by WHO in September 2016 and Botswana, will receive one more transitional grant from the Global Fund. The Philippines submitted their final proposal for funding in the first quarter of 2017 together with a transition plan for sustainable financing. Several other countries are approaching one or more donor eligibility thresholds in the next few years. Although the majority of funding in these countries comes from domestic sources, DAH still plays an important role in the delivery of health interventions, particularly to vulnerable populations that are often underserved by the government health system. Donors such as the Global Fund will need to continue to prioritize these populations to deliver on its 2017–2022 Global Fund Strategy, which aims to achieve progress toward a world free of the burden of HIV/AIDS, tuberculosis, and malaria.

The Global Fund continued to provide the largest source of DAH to malaria-endemic countries accounting for over 90% of all external financing. It is not possible to unpack donor contributions specifically to malaria disbursed by the Global Fund, however, in general, the US government provides 35% of all funding, the United Kingdom, 16%, France, 9% and non-official sources including foundations and charities, 6%. A more diverse set of donors including the World Bank and various bilateral donors played a larger role in the malaria agenda prior to the establishment of the Global Fund. For example, Australia played a major role in funding malaria control in the Pacific Islands; however, this funding has been drastically reduced since with the creation of the Department of Foreign Affairs and Trade replacing Australian Aid whose new Health for Development Strategy 2015–2020 [9] focuses on health as development with little on disease-specific funding.

Across the 35 countries included in this review, GHE almost doubled between 2000 and 2010, ultimately resulting in about $249 million in 2014 (excluding South Africa as an outlier). In most countries, the upward GHE trend between 2008 and 2014 has been maintained or increased. Nine countries included in the review (Algeria, El Salvador, Guatemala, Malaysia, Mexico, Panama, Paraguay, ROK, Saudi Arabia) are entirely domestically financed.

DAH was disaggregated into 13 service delivery areas allowing for cross-country and regional comparisons. The observed trends in spending or allocation by service delivery area are not uniform or consistent with epidemiological profiles or regional policies demonstrating the need for greater emphasis on allocative efficiency. Vector control, mostly bed nets continues to be the largest cost driver across all regions, followed predominately by treatment costs.

Thirty-one of 35 countries spent less than 10% of their malaria DAH funding on surveillance, a key malaria elimination intervention between 2010 and 2013. The ratio of DAH expenditure on diagnosis versus treatment increased after 2008 reaching a 50% split in most countries by 2013 bringing countries closer to compliance with WHO’s Test: Treat: Track policy. Notable exceptions are Honduras, Tajikistan, and Thailand with minimal expenditure on diagnosis. As actual cases decrease, expenditure on diagnosis is expected to be at least twice the spending on treatment. However, discrepancies between use of DAH for certain service delivery areas and strategy for malaria elimination could be explained by governments using DAH to fund allowable expenses and GHE to pay for the rest, for example procurement of diagnostics. Nevertheless, the analysis does raise the question on whether DAH is being spent on the most effective strategies for malaria elimination.

Morel and colleagues noted, “it is important to ask whether current interventions are used appropriately and what is the most cost-effective way to scale up activities to the levels needed” [19]. With declining DAH, available resource will need to be used more efficiently. This would include focusing the needs of the malaria programme on the most effective interventions coupled with better targeting of intervention delivery to strategic populations to maximize value-for-money and prevent drug and insecticide resistance and from available resources [18]. At the same time, there is a need to move donor funding for malaria control away from an input model that mostly focuses on the procurement and distribution of key inputs (most notably mosquito nets) towards more support for operational improvements, capacity building in programme management, improved disease and intervention surveillance as well as knowledge generation and sharing to strengthen the impact of elimination interventions.

The WHO Global Technical Strategy for Malaria estimated that USD 6.8 billion will be needed annually to reduce malaria related morbidity and mortality by 90% between 2015 and 2030 and projected gaps of more than half of this financing need. Although gains in health system efficiency can be used to make reduce the discrepancy between available finances and need, current trends suggest that many countries may face gaps in financing for malaria elimination. If increasing domestic health financing is the solution, countries will need to increase their own spending on malaria beyond historical trends. The expectation of the economic and health financing transition suggests that as countries develop they spend more on health than they did before. Of 35 currently low-income and middle-income countries, included in this review, 22 countries currently meet the Chatham House goal of spending 5% of GDP or $86 per capita on health [20].

There are several complementary ways for countries to fill the gap between needs and resources until government allocations catch up with the financing transition. The Addis Ababa Action Agenda calls on a number of resource mobilization efforts encompassing aid, domestic public resources, and support from the private sector. Many national governments are considering raising health budgets by improving the capacity to raise tax revenue including the implementation of Pigovian or sin taxes. In the Philippines, the Sin Tax Reform Bill, passed in 2012, increased taxes on tobacco and alcohol, generating USD 2.3 billion within 2 years increasing the Department of Health budget by 63% in 2015. This revenue has freed up resources, which would have otherwise been used for social protection of the poor and has trickled down for use for malaria and other diseases targeted for elimination.

Two other areas of resource mobilization which have had limited traction are better harnessing of private financing as well as innovative approaches, such as social impact bonds, airline and financial transactions taxes. Blended approaches which refer to the use of funds to leverage or de-risk private investment in development are increasingly being explored. Although there are no current estimates on their scale, these financing instruments have been used with success in other sectors within and outside of health and have the potential to catalyse future additional private sector support.

The Roll Back Malaria Action for Investment in Malaria (AIM) suggests that investment in malaria could deliver strong health benefits through fewer deaths and less illness that can be valued at over $49 billion. These benefits exceed investment costs by a factor of 40 over the period to 2030 [21]. Focused, advocacy at all levels is needed to reach key decision-makers in order to highlight the social and economic benefits of investing in malaria elimination and the risks of not doing so. In particular, emphasis on the threat of drug resistance in undermining success and posing a risk of regional health security is needed. Continued engagement is needed with governments to focus attention on increased domestic budgets.

This analysis has several limitations. Many of the DAH expenditures could not be allocated to specific interventions, therefore introducing a potential bias. In addition, the spending by governments could not be further disaggregated by intervention area and it is possible that DAH was spent on particular interventions due to co-financing of others through domestic sources. Estimates of domestic expenditures on malaria were obtained from sources which relied on self-reporting by countries with little triangulation of data and the findings should therefore be interpreted as such.

Nevertheless, the findings provide strong evidence on the uncertainty about the future availability of DAH in malaria elimination settings and the wide variation in support for malaria programmes by governments [21]. Many malaria-eliminating countries could risk facing funding gaps, which could be compounded if countries face funding cliffs with multiple donors phasing out simultaneously. These disruptions in service delivery could also confer negative cross-border externalities to neighbouring countries, compromising regional elimination targets and ultimately global eradication.

## Conclusion

Financing for malaria elimination is declining at a time when commitment to elimination will be crucial to paving the way to global malaria eradication. While government health expenditure has steadily increased in most countries, this increase has not been proportional to the rate of waning external financing, particularly in middle-income countries, increasing the risk of deadly and costly malaria resurgences. Notwithstanding, existing financing has not been used in the most cost-effective or efficient manner. Mechanisms to increase efficiency and value for money are urgently needed as well as further analysis on the extent to which expenditures are in line with the interventions recommended by the WHO. Innovative health financing mechanisms may provide a respite—until domestic financing is able to fill the gap created by diminishing donor resources.

## Abbreviations

AIM: Action and Investment to Defeat Malaria 2016–2030
APLMA: Asia Pacific Leaders Malaria Alliance
BMFG: Bill & Melinda Gates Foundation
CRS: Creditor Reporting System
DAH: Development Assistance for Health
E8: Elimination 8 (Block of 8 countries in southern Africa implementing regional approaches for elimination)
GHE: Government Health Expenditure (As Source)
Global Fund: The Global Fund to Fight AIDS, Tuberculosis and Malaria
GMS: Greater Mekong Subregion
GNI: Gross National Income
IHME: Institute for Health Metrics and Evaluation
IRS: indoor residual spraying
PMI: (United States) President’s Malaria Initiative
RAI: Regional Artemisinin-Resistance Initiative
RBM: Roll Back Malaria Initiative
ROK: Republic of Korea
SDG: Sustainable Development Goals
USD: US dollar
WHO: World Health Organization
WMR: World Malaria Report
